# FIE, a nuclear PRC2 protein, forms cytoplasmic complexes in *Arabidopsis thaliana*

**DOI:** 10.1093/jxb/erw373

**Published:** 2016-10-17

**Authors:** Moran Oliva, Yana Butenko, Tzung-Fu Hsieh, Ofir Hakim, Aviva Katz, Nechama I. Smorodinsky, Daphna Michaeli, Robert L. Fischer, Nir Ohad

**Affiliations:** ^1^Department of Molecular Biology and Ecology of Plant, The George S. Wise Faculty of Life Sciences, Tel Aviv University, Ramat Aviv, Tel Aviv, 69978, Israel; ^2^Department of Ornamental Horticulture and Plant Biotechnology, Agriculture Research Organization, The Volcani Center, PO Box 6, Beit Dagan, 50250, Israel; ^3^Department of Plant Sciences, Weizmann Institute of Science, Rehovot, 76100, Israel; ^4^Department of Plant Sciences, Faculty of Agriculture, Hebrew University of Jerusalem, Rehovot, 76100, Israel; ^5^Plants for Human Health Institute, and Department of Plant and Microbial Biology, North Carolina State University, Kannapolis, NC 28081, USA; ^6^The Mina and Everard Goodman Faculty of Life Sciences, Bar-Ilan University, Ramat-Gan, 5290002, Israel; ^7^Department of Cell Research and Immunology, The George S. Wise Faculty of Life Sciences, Tel Aviv University, Ramat Aviv, Tel Aviv, 69978, Israel; ^8^Department of Plant and Microbial Biology, University of California, Berkeley, CA 94720-3102, USA; ^9^The Manna Center Program for Food Safety and Security, Tel Aviv University, 69978,Israel

**Keywords:** Cytoplasm, FIE, MEA, PcG, Polycomb complex, PRC2.

## Abstract

Polycomb group (PcG) proteins are evolutionarily conserved chromatin modifiers that regulate developmental pathways in plants. PcGs form nuclear multi-subunit Polycomb Repressive Complexes (PRCs). The PRC2 complex mediates gene repression via methylation of lysine 27 on histone H3, which consequently leads to chromatin condensation. In *Arabidopsis thaliana*, several PRC2 complexes with different compositions were identified, each controlling a particular developmental program.

The core subunit FIE is crucial for PRC2 function throughout the plant life cycle, yet accurate information on its spatial and temporal localization was absent. This study focused on identifying FIE accumulation patterns, using microscopy and biochemical approaches. Analysing endogenous FIE and transgenic gFIE–green fluorescent protein fusion protein (gFIE-GFP) showed that FIE accumulates in the nuclei of every cell type examined. Interestingly, gFIE-GFP, as well as the endogenous FIE, also localized to the cytoplasm in all examined tissues. In both vegetative and reproductive organs, FIE formed cytoplasmic high-molecular-mass complexes, in parallel to the nuclear PRC2 complexes. Moreover, size-exclusion chromatography and bimolecular fluorescence complementation assays indicated that in inflorescences FIE formed a cytoplasmic complex with MEA, a PRC2 histone methyltransferase subunit. In contrast, CLF and SWN histone methyltransferases were strictly nuclear. Presence of PRC2 subunits in cytoplasmic complexes has not been previously described in plants. Our findings are in agreement with accumulating evidence demonstrating cytoplasmic localization and function of PcGs in metazoa. The cytosolic accumulation of PRC2 components in plants supports the model that PcGs have alternative non-nuclear functions that go beyond chromatin methylation.

## Highlight

FIE, a WD-40 subunit of the Arabidopsis PRC2 complexes known to take part in H3K27 methylation of nuclear chromatin, is also localized in cytoplasmic complexes.

## Introduction

Polycomb group proteins (PcGs) are highly conserved chromatin modifiers that regulate key developmental pathways in plants (Kohler and Villar, 2008; Derkacheva and Hennig, 2014) and animals (Schwartz and Pirrotta, 2007, 2008), selectively controlling temporal and spatial expression of numerous genes. PcG proteins mediate repression of gene expression through an epigenetic mechanism involving methylation of histone H3 at lysine 27, which subsequently leads to chromatin remodeling and condensation (Bouyer *et al.*, 2011; Kim *et al.*, 2012; Ikeuchi *et al.*, 2015; Horst *et al.*, 2016). In *Arabidopsis thaliana*, PcGs were found to play a major role in regulating various developmental processes, including the transition from the gametophytic to sporophytic phase, embryogenesis, and organogenesis, as well as the transition from the vegetative to reproductive phase and flower development. As much as 20–35% of the genes in Arabidopsis are potentially regulated by PcG proteins (Zhang *et al.*, 2007; Turck *et al.*, 2007; Bouyer *et al.*, 2011; Kim *et al.*, 2012).

Several multi-subunit Polycomb repressive complexes (PRCs) are known to mediate different steps in transcription silencing. In metazoans, at least three distinct PcG complexes were identified: PRC2, Polycomb-like PRC2 (Pcl-PRC2) and PRC1 (Muller and Verrijzer, 2009). The PRC2 complex, conserved in both animals (Korf *et al.*, 1998; Tie *et al.*, 2001; Poux *et al.*, 2001) and plants (Goodrich *et al.*, 1997; Grossniklaus *et al.*, 1998; Luo *et al.*, 1999; Ohad *et al.*, 1999), is responsible for initiating gene silencing by catalysing trimethylation of H3K27 on target loci (Czermin *et al.*, 2002; Muller *et al.*, 2002; Cao *et al.*, 2002, 2008; Kuzmichev *et al.*, 2002; Bouyer *et al.*, 2011; Kim *et al.*, 2012). Four core components of the PRC2 complex were identified in *Drosophila*: enhancer of zeste (E(z)); extra sex comb (ESC); suppressor of zeste 12 (Su(z)12) and the nucleosome remodeling factor 55-kDa (p55) (Martinez-Balbas *et al.*, 1998; Czermin *et al.*, 2002; Muller *et al.*, 2002).

In Arabidopsis, PRC2 complexes comprise homologs of the *Drosophila* E(z), namely CURLY LEAF (CLF) (Goodrich *et al.*, 1997), SWINGER (SWN) (Chanvivattana *et al.*, 2004) and MEDEA (MEA) (Grossniklaus *et al.*, 1998; Luo *et al.*, 1999). These proteins catalyse the methylation of H3K27 (a histone methyltransferase; HMTase) via the SET (Su(var) E(z) Thritorax) domain (Czermin *et al.*, 2002; Muller *et al.*, 2002). EMBRYONIC FLOWER 2 (EMF2) (Yoshida *et al.*, 2001), VERNALIZATION 2 (VRN2) (Gendall *et al.*, 2001) and FERTILIZATION INDEPENDENT SEED 2 (FIS2) (Luo *et al.*, 1999; Chaudhury *et al.*, 2001) are homologs of the Su(z)12 protein, a C2H2 zinc-finger protein that binds the HMTase subunit via the VEFS domain (Birve *et al.*, 2001). MULTI-SUBUNIT SUPPRESSOR OF IRA (MSI) 1–5 are homologs of the p55 protein (Ach *et al.*, 1997; Kenzior and Folk, 1998; Hennig *et al.*, 2003; Kohler *et al.*, 2003; Pazhouhandeh *et al.*, 2011; Derkacheva *et al.*, 2013). Contrary to all other PRC2 subunits, the Arabidopsis homolog of the *Drosophila* ESC WD-40 protein (Czermin *et al.*, 2002), termed FERTILIZATION INDEPENDENT ENDOSPERM (FIE), has a single copy gene (Ohad *et al.*, 1999; Kohler *et al.*, 2003; Derkacheva and Hennig, 2014).

Genetic, molecular and biochemical evidence demonstrated that at least three PRC2 complexes, harboring different catalytic and zinc-finger subunits, likely exist in Arabidopsis, each controlling a particular developmental program (Hsieh *et al.*, 2003; Chanvivattana *et al.*, 2004; De Lucia *et al.*, 2008; Bouyer *et al.*, 2011; Butenko and Ohad, 2011; Kim *et al.*, 2012). The reproductive FIS2–PRC2 complex, harboring MEA as its HMTase subunit, is implicated in regulating female gametophyte and seed development (Ohad *et al.*, 1996; Chaudhury *et al.*, 1997; Grossniklaus *et al.*, 1998; Kiyosue *et al.*, 1999; Kohler *et al.*, 2003; Guitton *et al.*, 2004). The vegetative EMF2–PRC2 complex, harboring CLF and/or SWN HMTases, suppresses premature transition from the vegetative to the reproductive stage and takes part in regulating floral organ development (Yang *et al.*, 1995; Chanvivattana *et al.*, 2004; Jiang *et al.*, 2008; Kim *et al.*, 2010). A second vegetative complex VRN2–PRC2, also harboring CLF and/or SWN HMTases, as well as VERNALIZATION INSENSITIVE 3 (VIN3), regulates flowering time mediated by vernalization (Chandler *et al.*, 1996; Gendall *et al.*, 2001; Wood *et al.*, 2006; De Lucia *et al.*, 2008). Both vegetative PRC2 complexes have been recently shown to take part in repressing dedifferentiation of root cells into embryos (Ikeuchi *et al.*, 2015).

Since FIE is encoded by a single-copy gene in Arabidopsis, it is probably an essential component of all PRC2 complexes that regulate different aspects of the plant life cycle (Katz *et al.*, 2004). The FIE protein was shown to interact with each of the HMTase subunits: MEA (Luo *et al.*, 2000; Yadegari *et al.*, 2000; Kohler *et al.*, 2003; Bracha-Drori *et al.*, 2004), CLF (Katz *et al.*, 2004; Mosquna *et al.*, 2009), and SWN (Luo *et al.*, 2000; Chanvivattana *et al.*, 2004), as well as with the MSI1 subunit (Kohler *et al.*, 2003). FIE was shown to regulate the female gametophyte and seed development (Ohad *et al.*, 1996; Chaudhury *et al.*, 1997), to establish the anterior–posterior polar axis in the endosperm (Sorensen *et al.*, 2001) and to inhibit endosperm overproliferation (Grossniklaus *et al.*, 1998; Luo *et al.*, 1999; Ohad *et al.*, 1999; Kohler *et al.*, 2003; Wang *et al.*, 2006). Accordingly, mutants impaired in FIE function were shown to develop seed-like structures containing juvenile endosperm in the absence of fertilization, and arrested embryos surrounded by overproliferated endosperm when fertilization took place, leading to seed abortion in both cases (Ohad *et al.*, 1996; Chaudhury *et al.*, 1997; Grossniklaus *et al.*, 1998; Luo *et al.*, 1999; Kiyosue *et al.*, 1999; Guitton *et al.*, 2004). FIE also partakes in regulating the transition from embryonic to seedling phase (Bouyer *et al.*, 2011), while during the vegetative stage FIE is required for regulating leaf development and flowering time (Kinoshita *et al.*, 2001; Katz *et al.*, 2004; Chanvivattana *et al.*, 2004). *fie*-cosupressed mutants, which lose *FIE* expression during postembryonic stages, display multiple morphological and developmental aberrations, such as loss of apical dominance, curled leaves and precocious flowering (Katz *et al.*, 2004). Additionally, leaves and flower organs in these mutants undergo homeotic transformation to carpel-like structures, demonstrating the vital role of FIE in regulating organogenesis (Yoshida *et al.*, 2001; Kinoshita *et al.*, 2001; Katz *et al.*, 2004). Furthermore, *fie* null mutants display a progressive loss of cell differentiation states after germination, resulting in the formation of a disorganized callus-like plant body (Bouyer *et al.*, 2011).

The fundamental function of FIE in regulating developmental programs along the plant life cycle, including phase transition from the gametophytic to the sporophytic phase, is highly conserved through plant evolution (Butenko and Ohad, 2011). In the moss *Physcomitrella patens*, the single-copy *PpFIE* gene controls cell proliferation and differentiation in both the recessive sporophytic and the dominant gametophytic stages of the plant life cycle (Mosquna *et al.*, 2009; Okano *et al.*, 2009; Pereman *et al.*, 2016; Horst *et al.*, 2016). While PpFIE protein was mostly evident in cells undergoing cell-fate transition (Mosquna *et al.*, 2009), in flowering plants transcription of the *FIE* gene is ubiquitous (Springer *et al.*, 2002; Danilevskaya *et al.*, 2003; Katz *et al.*, 2004; Zimmermann *et al.*, 2004; Hermon *et al.*, 2007; Rodrigues *et al.*, 2008; Luo *et al.*, 2009; Kapazoglou *et al.*, 2010). While in dicots *FIE* is a single copy gene, two *FIE*-like genes were identified in the genomes of several monocot species (Springer *et al.*, 2002; Danilevskaya *et al.*, 2003; Luo *et al.*, 2009). One *FIE*-like gene is expressed ubiquitously, while the other is expressed only in the endosperm (Springer *et al.*, 2002; Danilevskaya *et al.*, 2003; Hermon *et al.*, 2007; Luo *et al.*, 2009; Butenko and Ohad, 2011).

Though FIE was shown to be essential throughout the Arabidopsis life cycle, little is known regarding its accumulation pattern. To visually monitor FIE accumulation during the Arabidopsis life cycle, lines bearing a transgene expressing an FIE–green fluorescent protein (GFP) fusion protein from the endogenous *FIE* promoter were generated. FIE-GFP was able to fully complement *fie* mutant phenotype. As expected, FIE-GFP and endogenous FIE were found to be nuclear localized in all tissues tested. However, a significant fraction of FIE-GFP, as well as of the endogenous FIE, was localized in the cytoplasm. Moreover, MEA HMTase protein was found to interact with FIE in the cytoplasm and to form high-molecular-mass protein complexes. Taken together, these results suggest that, in addition to their nuclear function of maintaining chromatin transcriptional regulation, plant PRC2 complexes may have novel distinct functions in the cytoplasm.

## Materials and methods

### Plant materials and growth conditions

The Arabidopsis (*Arabidopsis thaliana*) Columbia-0 (Col-0) and Landsberg *erecta* (L*er*) cultivars were used as the wild-type (WT). Mutants used in this study were: *fie-1*^*+/−*^ *547* (Kinoshita *et al.*, 2001), *clf-2* (Goodrich *et al.*, 1997; CS8853 in the Arabidopsis stock center), *clf* SALK mutant 006658, *swn-3* (Chanvivattana *et al.*, 2004; Salk 050195.6) and *mea f644* (Kiyosue *et al.*, 1999).

Plants were grown in Percival incubators at 22 ± 2°C under white fluorescent illumination (100 μE m^−2^ s^−1^) in 16 h light–8 h dark cycles (long day). All seeds were sown on half-strength Murashige and Skoog (MS) medium (Murashige and Skoog, 1962) supplemented with 1% sucrose and B5 vitamins, under selection conditions when needed.

### Generation of antibodies against plant PRC2 proteins FIE, MEA, CLF and SWN

Antibodies were raised in New Zealand White rabbits. αMEA antibodies were generated against a polypeptide corresponding to amino acids (aa) 285–300 of MEA protein. The αMEA antibody recognizes the MEA protein of the expected 97 kDa size, and in addition polypeptides with smaller molecular mass (Fig. 5C, upper panel, arrows). αCLF antibodies were generated against a polypeptide corresponding to aa 1–130 of the CLF protein. αSWN antibodies were generated against polypeptides corresponding to aa 1–122 and to aa 552–568 of the SWN protein. All three antibodies were shown to detect the endogenous proteins at their expected sizes from extracts from wild-type plants, but not in respective mutants (see Supplementary Fig. S1 at *JXB* online). Generation and characterization of αFIE no. 61 antibodies was previously described (Katz *et al.*, 2004).

### Protein extraction and immunoblot analyses

#### Native extraction of cytosolic soluble proteins

Extraction of soluble cytosolic proteins was performed as previously described (Katz *et al.*, 2004). Plant tissues were ground to a fine powder with liquid nitrogen and then homogenized in protein extraction buffer (100 mM Tris pH 7.2, 10% sucrose, 5 mM MgCl_2_, 5 mM EDTA, 40 mM β-mercaptoethanol, and Complete Protease Inhibitor Cocktail (Roche)) at a ratio of 1 ml to 1 g tissue (initial weight). The homogenate was centrifuged twice at 14 000 *g* for 10 min and the supernatant was collected. The resulting native cytosolic protein extract was further used for sedimentation and size-exclusion chromatography assays, or mixed with sample buffer and analysed by protein immunoblotting as previously described (Katz *et al.*, 2004).

#### Differential ultracentrifugation

Native cytosolic protein extract, prepared from 1 g of fresh tissue, was pelleted using a mini-ultracentrifuge (Beckman) at 150 000 *g* and 4 ºC for 1 h. In these conditions, all cell organelles, large protein complexes, non-soluble proteins, and cell membranes precipitate to the pellet, whereas the soluble cytosolic proteins remain in the supernatant. The soluble fractions were collected and analysed by protein immunoblotting using αFIE or αMEA antibodies.

#### Nuclei enrichment

Plant tissue (1–15 g) was ground to a homogeneous fine powder with liquid nitrogen then homogenized in ice-cold Buffer HG1 (1 M hexylene glycol, 0.01 M Pipes pH 7.0, 0.01 M MgCl_2_, 0.2% Triton X-100, 1 µM ZnSO_4_, 0.01 M KCl, 5 µM dithioerythritol (DTE), 2 mM phenylmethylsulfonyl fluoride (PMSF) and Complete Protease Inhibitor Cocktail (Roche)) at a ratio of 10 ml to 1 g of tissue (initial weight). The suspension was filtered stepwise through 100 nm, 50 nm and 20 nm mesh, and then centrifuged for 10 min at 2000 *g* and 4 ºC. The pellet was washed twice in ice-cold Buffer HG2 (0.5 M hexylene glycol, 0.01 M Pipes pH 7.0, 0.01 M MgCl_2_, 0.2% Triton X-100, 0.01 M KCl, 5 µM DTE and Complete Protease Inhibitor Cocktail (Roche)) at a ratio of 5 ml to 1 g of tissue (initial weight), then centrifuged twice for 10 min at 5000 *g* and 4 ºC. Nuclei-enriched pellet was collected for further extraction steps or stored in 50% glycerol at –80 ºC.

#### Chromatin protein extraction

Extraction of chromatin proteins was performed on a nuclei-enriched fraction. For immunoblotting analyses, the nuclei-enriched pellet was incubated for 20 min at room temperature (RT) in equal volume (v/v) of ice-cold denaturative nuclei lysis buffer (50 mM Tris pH 7.5, 10 mM EDTA, 1% sodium dodecyl sulfate (SDS) and 0.2 mM PMSF and Complete Protease Inhibitor Cocktail (Roche)) with 1–2 μl DNase1 (Sigma-Aldrich) per sample. Extract was cleared by centrifugation for 10 min at 14 000 *g* and RT and the supernatant collected.

For size-exclusion chromatography analyses, the nuclei-enriched pellet was resuspended in equal volume (v/v) of ice-cold native chromatin extraction buffer (250 mM NaCl, 50 mM Hepes, 5 mM EDTA, 0.1 mM PMSF, 0.5 mM DTE, 5 mM MgCl_2_ and Complete Protease Inhibitor Cocktail (Roche)) with 1–2 μl DNase1 (Sigma-Aldrich) per sample. The extract was flash-frozen in liquid nitrogen to disrupt the nuclear envelope and then incubated on an orbital mixer for 30 min at 4 ºC. The resulting nuclei lysate was centrifuged for 10 min at 14 000 *g* at RT and the supernatant was collected. The pellet was extracted again as described above, and the supernatant was combined with the previous one.

### Size-exclusion chromatography

Size-exclusion chromatography (SEC) was performed on native cytoplasmic or chromatin-associated protein extracts from either rosette leaves of 17-day-old plants or inflorescences (flowers after anthesis). Total protein concentration was determined using the Bradford assay for cytoplasmic extracts, but not for low-protein-content nuclear extracts. Chromatography was carried out using an ÄKTA fast-performance liquid chromatograph (FPLC) (Amersham Pharmacia Biotech) at 4 ºC. Extracts (up to 0.5 ml and/or 500 mg of protein) were applied to an HR 10/30 Superose 6 column (Amersham Pharmacia Biotech) equilibrated in extraction buffer. Proteins were eluted at 0.3 ml min^–1^ under maximum pressure of 1 MPa. Fractions of 1 ml for rosette leaves and of 0.5 ml for inflorescences were collected. Size of eluted proteins was calibrated using protein size marker standards (Amersham Pharmacia Biotech). Proteins from each fraction were concentrated using 10 μl of Strata Clean Resin Beads (Stratagene) by incubating for 1 h at 4 ºC. Proteins were released from the beads by boiling for 5 min in sample buffer and analysed by immunoblotting.

### Generation of transgenic *ProFIE:FIE*_*gDNA*_*-GFP* plants

#### Construction of transformation vector and Arabidopsis transformation

The *gFIE-GFP* genomic construct, spanning a 4991 bp *FIE* (At3g20740.1) genomic region (between tair10 chr3 position 7248538-7253528, bottom strand), was produced by joining three DNA fragments with partial overlapping sequences to facilitate fusion by PCR reaction: 1274 bp of FIE promoter sequences and 3188 bp of coding sequences minus the stop codon, a *sGFP* fragment, and 529 bp *FIE* 3′-end sequences (from the stop codon to an *Xho*I site). The promoter and coding sequences were amplified from WT genomic DNA with primers ‘FIEgf3873’ (5′GGGAGCTTCAGATGTTCTATTAAGATACCC3′) and ‘FIENcoI’ (5′CACCATGGCTCCGC CACCTCCGCCACCCTT GGTAATCACGTCCCAGCG3′). The *sGFP* fragment was amplified from pBI-sGFP plasmid DNA using primers ‘FIE/sGFPlf’ (5′AA GGGTGGCGGAGGTGGCGGAGCCATGGTGAGCAAG3′) and ‘sGFP/FIElr’ (5′ACAAGACTCAGACCGCTACTTGTACA GCTCGTCCATGCCGTGAG3′). The *FIE* 3′-end fragment was amplified using ‘sGFP/FIElf’ (5′CTGTACAAGTAGCGGTCTGAG TCTTGTAGGAATTGATGAATTAGGAG3′) and T7 primers from a pBluescript plasmid template that contains the *Cla*I/*Xho*I 4991 bp *FIE* genomic region. The three PCR fragments were purified, mixed at an equal molar ratio, and amplified with the FIEgf3873 and T7 primers to obtain the full length *gFIE-GFP* fragment, which was subsequently cloned into the pBI-sGFP binary vector as a blunt-ended fragment, replacing the *sGFP-Nos* terminator sequences. The sequence-verified construct was introduced into *Agrobacterium tumefaciens* strain GV3101 pMP90 (Koncz and Schell, 1986).

Transformation of Arabidopsis Col-0 was performed by the floral dip method (Clough and Bent, 1998). Twenty independent kanamycin-resistant T1 plants were obtained and confirmed to contain the *gFIE-GFP* insert by PCR.

### Testing the functionality of gFIE-GFP by complementing the *fie+/–* phenotype

The complementation assay, adapted from (Kinoshita *et al.*, 2001), measures the ability of the *ProFIE:FIE*_*gDNA*_*-GFP* transgene to complement the seed abortion phenotype associated with inheritance of a maternal mutant *fie-1* allele. Independent female kanamycin-resistant T1 *ProFIE:FIE*_*gDNA*_*-GFP* plants, each representing a distinct transgenic line, were crossed with *FIE*/*fie* heterozygous males. The resulting F1 seeds were germinated on kanamycin (50 mg l^–1^) to select for the *ProFIE:FIE*_*gDNA*_*-GFP* transgene, and resistant plants were genotyped via PCR to identify *FIE/fie* heterozygous plants. Self-pollinated siliques were examined for percentage of aborted seeds. In plants heterozygous for *fie-1*/*FIE* and hemizygous for the *ProFIE:FIE*_*gDNA*_*-GFP* transgene, full complementation by gFIE-GFP is expected to result in 25% seed abortion, instead of 50% observed in control *fie-1*/*FIE* heterozygous plants.

Molecular analysis for genotyping the endogenous *FIE* locus was performed by a two-step PCR reaction to amplify a sequence spanning the point mutation in the *fie-1* allele. The PCR product was then subjected to Tsp 504I, a restriction enzyme that specifically recognizes the *fie-1* mutated sequence, but not the wild-type sequence.

### Bimolecular fluorescence complementation analysis

Protein–protein interaction in plants was examined by Bimolecular Fluorescence Complementation (BiFC) assay (Bracha-Drori *et al.*, 2004; Ohad *et al*., 2007). *FIE*, *MEA*, *CLF* and *SWN* full-length cDNAs were cloned into the *Spe*I site of pSY 751 and pSY 752 binary vectors, which contain the N-terminal (YN) and the C-terminal (YC) fragments of yellow fluorescent protein (YFP), respectively (Bracha-Drori *et al.*, 2004; Mosquna *et al.*, 2009). Equal concentrations of *Agrobacterium tumerfaciens* strain GV3101/pMp90 containing the plasmids of interest were transiently coexpressed in Arabidopsis cotyledons or in *Nicotiana benthamiana* leaves. The procedure of transient transformation in Arabidopsis seedlings was based on protocols described by Marion *et al.* (2008) and Li *et al.* (2009) with the following modifications: Arabidopsis seedlings were grown on MS-agar in standard six-well plates, 20–30 seeds per well, for 5–6 days until the cotyledons emerged. *Agrobacterium* cells harboring the appropriate plasmids were grown at 28 ºC in Induction medium (Bracha-Drori *et al.*, 2004) supplemented with antibiotics and 0.2 mM acetosyringone, to give a final OD_600_ of 2–4. For transformation, the bacterial cultures were diluted in Induction medium to OD_600_ of 1–1.5, mixed in 1:1 ratio and poured on the seedlings until covering them completely. Open plates were placed in a vacuum desiccator jar, and infiltration was performed by applying a vacuum (80 psi) for 2 min ×3. Then, the infiltration medium was removed and the plates were returned to a growth chamber. Protein expression was examined 48–72 h following infiltration by confocal laser scanning microscopy (CLSM).

*N. benthamiana* leaves were co-transformed with YC-HA-FIE and YN-GG-MEA constructs by leaf infiltration as described in Bracha-Drori *et al.* (2004). Protein expression was examined 24–48 h following injection by CLSM.

### Plasmolysis assay

To induce plasmolysis, leaves or whole Arabidopsis seedlings were submerged in 0.8 M NaCl solution for several minutes. The leaf samples were mounted on microscope slides in the same solution and analysed by CLSM.

### Confocal microscopy

CLSM was performed using a Leica TCS-SL as previously described (Bracha-Drori *et al.*, 2004). Image analysis was performed using ImageJ 1.45s (NIH, USA; Abramoff *et al.*, 2004; http://imagej.nih.gov/ij/) and Adobe Photoshop 7.0.

### Generation of transgenic MEA-3HA line

MEA cDNA (At1g02580) sequence was amplified without the stop codon and ligated in-frame to the 3′ end 3×hemagglutinin (3×HA) epitope tag, followed by a TGA stop codon, into a modified binary Ti plasmid pZP111. The resulting *pZP111-35S:MEA-3×HA-T(Nos*) construct was verified by sequencing and introduced into *Agrobacterium tumefaciens* strain GV3101 pMP90 (Koncz and Schell, 1986). Transformation of Arabidopsis Col-0 was performed by the floral dip method (Clough and Bent, 1998). The MEA-3×HA protein expressed efficiently in inflorescences and was used for co-immunoprecipitation (Co-IP) assay.

### Co-immunoprecipitation of cytoplasmic proteins

The cytosolic fraction was isolated from inflorescences of MEA-3HA plants or *N. benthamiana* leaves using the method for native extraction of cytosolic soluble proteins. To remove insoluble material, extracts were centrifuged for 30 min at 20 000 *g* and 4°C. The supernatants were incubated with αHA (Covance, Cat No. MMS-101R; 1:100) or αGlu-Glu (Covance, Cat No. MMS-115R; 1:1000) monoclonal antibodies for 12 h at 4 ºC and then agitated with 25 μl protein G Sepharose beads (4-fast flow, Amersham Pharmacia Biotech) for 1 h to precipitate the antibody–antigen complexes. The beads were precipitated by centrifugation at 10 000 *g* and 4 ºC for 1 min, washed four times with 1 ml of phosphate-buffered saline then eluted from the beads by boiling them in sample buffer for 5 min. Equal volumes of immunoprecipitated and unbound proteins were used for immunoblotting. The membranes were then incubated with αGFP (Covance, Cat No. MMS-118P; 1:1000), αHA (1:3000) or αFIE antibodies.

## Results

*FIE* mRNA was previously shown to accumulate in a wide range of tissues in Arabidopsis, encompassing both vegetative and reproductive organs (Kiyosue *et al.*, 1999; Yadegari *et al.*, 2000; Katz *et al.*, 2004; Zimmermann *et al.*, 2004). *FIE* transcript was detected during ovule development and after fertilization in both the embryo and endosperm (Kiyosue *et al.*, 1999).

### FIE protein accumulates in all Arabidopsis tissues and organs

**Fig. 1.**
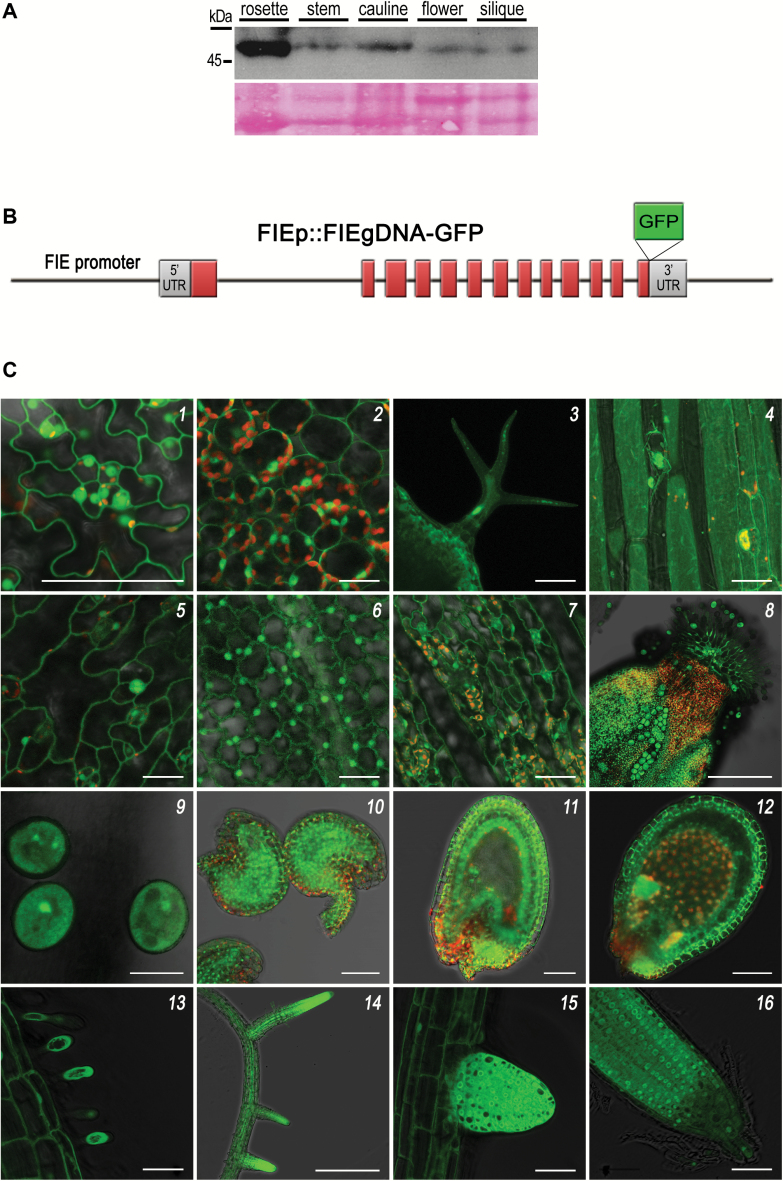
FIE protein accumulates in all Arabidopsis tissues and organs. (A) Detection of endogenous FIE protein in Arabidopsis tissues. A similar amount of nuclear chromatin protein extracts from rosette, stems, cauline, and inflorescence of 20-day-old wild-type plants and siliques from 6–10 days after pollination (DAP) were separated by SDS-PAGE and then immunodetected using αFIE antibodies. Protein size marker is indicated on the left. Ponceau staining was used to assess equal sample loading. (B) A scheme of the *ProFIE:FIE*_*gDNA*_*-GFP* transgene construct (not drawn to scale). Red boxes represent exons. (C) gFIE-GFP chimeric protein from line no. TH142-1 is observed in reproductive and vegetative tissues and organs. Each image was taken from a single focal plane, unless otherwise indicated. Each panel shows a merge of GFP epifluorescence (green) and the corresponding chloroplast autofluorescence (red) and/or a bright-field image. (1) Rosette leaf, abaxial epidermis; scale bar (SB): 50 μm. (2) Rosette leaf, mesophyll cells; SB: 25 μm. (3) Trichome on the surface of a rosette leaf, Z-stack overlay; SB: 50 μm. (4) Hypocotyl, Z-stack overlay; SB: 50 μm. (5) Cauline leaf, abaxial epidermis; SB: 25 μm. (6) Petal; SB: 25 μm. (7) Sepal; SB: 50 μm. (8) Carpel; SB: 250 μm. (9) Pollen; SB: 20 μm. (10) Unfertilized ovules; SB: 50 μm. (11) Developing seed containing a four cell-stage embryo; SB: 50 μm. (12) Developing seed containing a heart-stage embryo; SB: 100 μm. (13) Main root, root hairs; SB: 50 μm. (14) Main and lateral roots; SB: 500 μm. (15) Budding lateral root; SB: 50 μm. (16) Main root tip, quiescent center; SB: 50 μm.

As mRNA accumulation does not necessary correlate with presence of the protein product, FIE protein accumulation during plant development was characterized, using specific αFIE antibodies (Katz *et al.*, 2004). Immunoblot analysis of nuclear protein extracts from different plant tissues demonstrated that FIE was present in all tissues tested, including juvenile rosette leaves, cauline leaves, flowering stems, inflorescences and siliques (Fig. 1A).

To resolve the patterns of FIE accumulation at the sub-organ level, a construct constituting a genomic fusion between FIE and GFP under regulation of the native FIE promoter was used to generate transgenic plants. To ensure that FIE-GFP spatiotemporal expression mimics the endogenous FIE pattern, the entire endogenous *FIE* locus (At3g20740) was incorporated into the transgene, including all the introns, promoter, and genomic sequence downstream of the STOP codon (Fig. 1B). This construct, designated *ProFIE:FIE*_*gDNA*_*-GFP*, was introduced into wild-type Col-0 plants, giving rise to several transgenic lines.

To reveal whether the chimeric protein, designated gFIE-GFP, is fully functional, a complementation assay was performed, in which the ability of the transgene to rescue the *fie* seed abortion phenotype was tested. Independent *ProFIE:FIE*_*gDNA*_*-GFP* transgenic lines, each carrying the transgene at a single hemizygous locus in a wild-type background, were crossed to heterozygous *fie-1* mutant plants. T1 plants bearing the *fie-1* allele were identified based on seed abortion phenotype, and the percentage of aborted seeds in the siliques was determined. *fie* is a female gametophytic lethal mutation and therefore heterozygous *FIE/fie* plants abort ~50% of their seeds (Ohad *et al.*, 1996; Katz *et al.*, 2004). Accordingly, a single *ProFIE:FIE*_*gDNA*_*-GFP* transgenic allele introduced into the *FIE/fie* background should reduce the seed abortion rate to 25% if full complementation occurs. Such reduction was detected in plants derived from lines 141 and 142 (χ^2^ test, *P*<0.05), indicating full complementation of the seed abortion phenotype (Table 1). Furthermore, mature *fie*/*fie gFIE-GFP*/*gFIE-GFP* plants exhibited wild-type phenotype (see Supplementary Fig. S2), and none of the developmental aberrations observed in FIE-deficient mutants (Katz *et al.*, 2004; Bouyer *et al.*, 2011), thus confirming that the gFIE-GFP protein is biologically functional. All further analyses were performed on progeny of line 142, termed line TH142, which is homozygous for the *ProFIE:FIE_gDNA_-GFP* transgene in wild-type Col-0 background.

**Table 1.** *Complementation assay of FIE/fie using pFIE:FIE_gDNA_-GFP plants* Percentage seed abortion in self-pollinated heterozygous *FIE*/*fie* plants hemizygous for the *ProFIE:FIE*_*gDNA*_*-GFP* transgene.

Self-pollinated siliques		Seed segregation				Plant genotype (*FIE* allele)	*P*-value of χ^2^
Line no.	Plant no.	Normal	Aborted	Total	Percentage abortion		
TH127	8	97	57	154	37.01	*FIE/fie*	1 × 10^–3^
	10	97	60	157	38.22	*FIE/fie*	3 × 10^–3^
TH141	1	205	91	296	30.74	*FIE/fie*	3 × 10^–11^
	6	173	82	255	32.16	*FIE/fie*	1 × 10^–8^
	7	223	88	311	28.30	*FIE/fie*	2 × 10^–14^
	8	160	55	215	25.58	*FIE/fie*	8 × 10^–12^
TH142	1	144	49	193	25.39	*FIE/fie*	4 × 10^–13^
	3	138	41	179	22.91	*FIE/fie*	5 × 10^–11^
	4	112	33	145	22.76	*FIE/fie*	8 × 10^–13^

The distribution of gFIE-GFP fluorescence in transgenic lines was analysed by CLSM. The gFIE-GFP fusion protein was clearly detected in the nucleus of all cells examined, throughout the plant life (Fig. 1, Supplementary Fig. S3). These included vegetative tissues, namely rosette leaves, epidermis, trichome, and hypocotyl (Fig. 1C,1–4); cauline leaves and reproductive organs (Fig. 1C, 5–8); pollen and ovule (Fig. 1C, 9–10); embryo and developing seed (Fig. 1C, 11–12); and roots and meristematic apexes (Fig. 1C, 13–16). Thus, the accumulation pattern of the gFIE-GFP overlapped with the known expression pattern of *FIE* mRNA as reported so far (Kiyosue *et al.*, 1999; Yadegari *et al.*, 2000; Katz *et al.*, 2004; Zimmermann *et al.*, 2004). Collectively, these data indicate that FIE is indeed widely accumulated throughout the entire Arabidopsis life cycle.

### FIE protein localizes to the cytoplasm

**Fig. 2.**
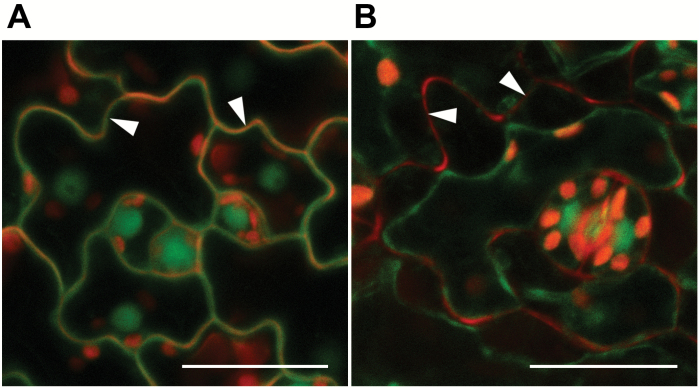
gFIE-GFP is localized to the cytoplasm. Laser scanning confocal microscopy imaging was employed following plasmolysis of Arabidopsis rosette leaf cells to determine the subcellular localization of the gFIE-GFP fusion protein (green). To visualize the cell walls, leaf samples were stained with propidium iodide (red). Each image was taken from a single focal plane. (A) Cells before plasmolysis. (B) Plasmolysed cells. Arrowheads indicate cell wall. Scale bar: 25 μm.

Interestingly, the fluorescence signal from the gFIE-GFP protein was not confined only to the nucleus, but was also clearly evident in the cytoplasm of all cells tested (Fig. 1C). In mature tissues, the intracellular GFP fluorescence was visualized as a thick band in the cell periphery, typical of cytoplasmic appearance in mature cells with large vacuoles (Fig. 1C, 1, 2, 4, 6, 7). In young cells with small vacuoles, such as cells located in budding lateral root, in which the cytoplasm is not pressed to the cell wall, gFIE-GFP protein was observed in the entire volume of the cell (excluding the vacuole) (Fig. 1C, 15–16).

A plasmolysis analysis performed on leaf epidermal cells of the transgenic plants established that the extranuclear gFIE-GFP protein localized to the cytosol, but not to the cell wall or vacuole (Fig. 2). As compared with non-treated cells (Fig. 2A), plasmolysed cells displayed a characteristic retraction of the plasma membrane from the cell wall, and gFIE-GFP fluorescence was detected within the wall-detached cytoplasm, but not in the cell wall or in the vacuole (Fig. 2B). The chloroplasts were clearly located on the inside of the GFP-labeled band (Fig. 2B), indicating that gFIE-GFP was not located in the tonoplast or in the vacuole. This suggested that the novel extranuclear compartment in which gFIE-GFP protein resides is the cytoplasm.

**Fig. 3.**
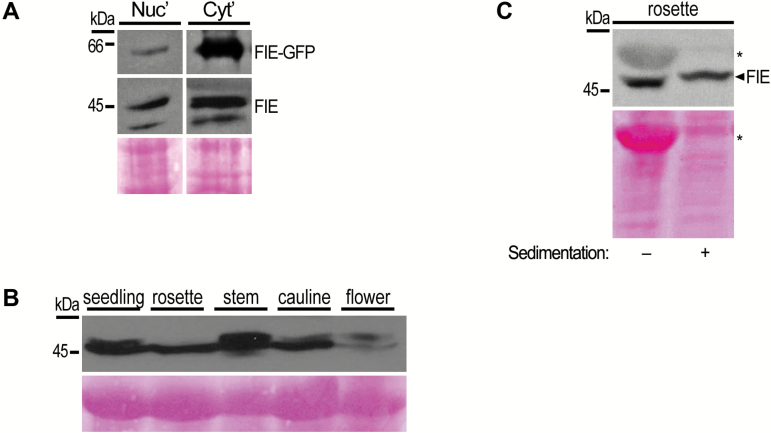
Endogenous FIE protein accumulates in the cytoplasm. (A) Nuclear (Nuc) and soluble cytoplasmic (Cyt) proteins were extracted from inflorescences of FIE-GFP plants, and analysed by SDS-PAGE followed by immunoblotting with αFIE and αGFP (Covance) antibodies. (B) Native cytoplasmic proteins were extracted from various tissues of wild-type Arabidopsis plants and equal amounts of samples were analysed by SDS-PAGE. The blot was probed with αFIE antibody. FIE was detected as a double band in all tissues, aside from rosette leaves. (C) Non-denaturated cytosolic protein extracts from rosette leaf tissue were sedimented by ultracentrifugation. Equal volumes from supernatant soluble fraction before and after (−/+) sedimentation were analysed by SDS-PAGE and probed with αFIE antibody. Absence of RuBisCO large chain protein band at ~53 kDa (marked with asterisk) following ultracentrifugation demonstrated successful sedimentation of large protein complexes. Ponceau staining in panels (A–C) was used to assess equal loading of samples.

As cytoplasmic localization of PRC2 proteins was not reported previously in plants, further characterization of this phenomenon was pursued. Cytosolic and nuclear protein fractions, extracted from various tissues of wild-type and gFIE-GFP plants, were analysed by immunoblotting for the presence of native and transgenic FIE proteins. Both the gFIE-GFP and the endogenous FIE proteins were detected in nuclear and in cytoplasmic fractions (Fig. 3A), which is in line with the gFIE-GFP fluorescence distribution (Fig. 1C). The purity of the cytosolic fraction was confirmed by the detection of actin only in the cytosolic fraction and H3K27me^2^ only in the nuclear fraction (see Supplementary Fig. S4A). Endogenous FIE, as well as gFIE-GFP, was detected in the cytoplasmic fractions in all examined tissues (Fig. 3B and Supplementary Fig. S4B), including seedlings, mature rosette leaves, stems, cauline leaves and inflorescences. Endogenous FIE was detected in two closely migrating forms with an estimated size of ~45 kDa (Fig. 3A, B), except in rosettes, suggesting that alternative forms of the FIE protein exist in the cytosol *in vivo*. This polymorphic appearance may be due to post-translational modification.

Further characterization of cytosolic FIE protein extracted from wild-type rosette leaves by ultracentrifugation revealed that FIE was mostly present in the soluble supernatant fraction (Fig. 3C). As this method sediments large protein complexes and insoluble organelles, it is likely that a large portion of FIE is present as a soluble protein in the cytosol.

### FIE forms a high-molecular-mass complex in the cytoplasm

**Fig. 4.**
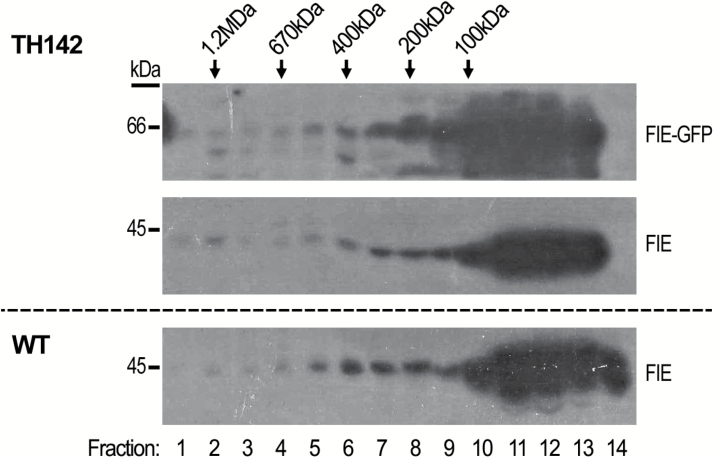
FIE forms high-molecular-mass complexes in the cytosol. Size-exclusion chromatography analysis of cytosolic proteins extracted from rosette leaves of 17-day- old seedlings of WT and TH142 (*ProFIE:FIE*_*gDNA*_*-GFP*) transgenic plants. The blots were immunoprobed with αGFP (top panel) and αFIE (middle and bottom panels) antibodies. Size markers (kDa) are indicated above relevant fractions. Large amounts of gFIE-GFP protein in fractions 10–14 above and below the main band are probably the result of large amounts of polypeptide. Migration of FIE protein in fractions 6–10 to an apparent lower molecular size band, as compared with other fractions, may result from the presence of a large amount of RuBisCO large chain (~53 kDa), as seen in Fig. 3C.

To examine whether cytosolic FIE forms complexes *in vivo*, cytoplasmic protein extracts from WT and gFIE-GFP transgenic plants were analysed by size-exclusion chromatography (SEC). Analysis of cytoplasmic protein extracts from rosette leaves showed FIE and gFIE-GFP proteins to be present in fractions 1–2 and 5–9, corresponding to high-molecular-mass proteins (Fig. 4), with molecular masses of ~1.2 MDa and 150–500 kDa, respectively. Monomeric gFIE-GFP and FIE were expected to elute between fractions 11 and 13, as their calculated masses are 66 and 45 kDa, respectively. Therefore, detection of FIE proteins in fractions representing larger molecular mass indicates that FIE takes part in a cytosolic protein complex. However, the majority of the cytoplasmic FIE eluted in the fractions corresponding to low molecular mass proteins (fractions 10–14), which is in agreement with FIE enrichment in the soluble fraction after sedimentation from rosette extracts (Fig. 3C).

A similar allocation of gFIE-GFP protein to fractions corresponding to high-molecular-mass complexes was observed in a parallel SEC analysis of nuclear extracts, purified from rosette leaves of a similar age (see Supplementary Fig. S5). Two HMTases, CLF and SWN, were detected in the same fractions with FIE (Supplementary Fig. S5), using specific antibodies generated in this study (Supplementary Fig. S1). This is in line with a previous report demonstrating that CLF, SWN and FIE take part in the same nuclear complex (Wood *et al.*, 2006).

### MEA interacts with FIE in the cytoplasm

**Fig. 5.**
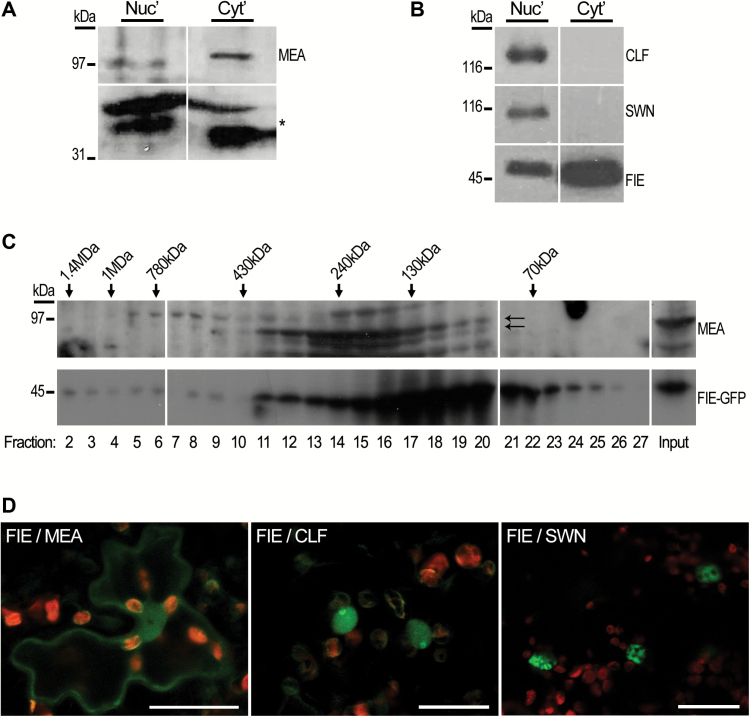
Subcellular localization of the interaction between FIE and PcG-SET domain proteins. (A) Equal amounts of nuclear (Nuc) and cytoplasmic (Cyt) protein extracts from WT inflorescences were analysed by SDS-PAGE and probed with αMEA antibodies. Equal loading was based on the presence of non-specific low-molecular-mass proteins in both samples (marked with asterisk). (B) Nuclear and cytoplasmic protein extracts of 14-day-old wild-type seedlings analysed by SDS-PAGE and probed with αCLF, αSWN and αFIE antibodies. CLF and SWN proteins were detected only in the nuclear fraction, while FIE was detected in both fractions. (C) Size-exclusion chromatography analysis of cytosolic proteins extracted from inflorescences (flowers after anthesis) of *ProFIE:FIE*_*gDNA*_*-GFP* transgenic plants. The blots were immunoprobed with αMEA (top lane) and αGFP (lower lane) antibodies. Arrows point to a 97 kDa MEA polypeptide (upper arrow), and non-specific faster migrating polypeptides (lower arrow). Size markers (kDa) are indicated above relevant fractions. (D) BiFC assay in Arabidopsis cotyledon leaves using YC-HA-FIE/YN-GG-MEA, YC-HA-FIE/YN-GG-CLF and YC-HA-FIE/YN-GG-SWN. Red signal, chloroplast autofluorescence; green signal, reconstituted-YFP fluorescence, resulting from the interaction. Scale bar: 20 μm.

To examine the possibility that the cytoplasmic FIE-containing complex may comprise additional known PRC2 subunits, nuclear and cytoplasmic protein extracts from wild-type Arabidopsis tissues were analysed by immunoblotting using specific anti-MEA, CLF and SWN antibodies. Surprisingly, endogenous MEA was identified in the cytoplasmic fractions from inflorescences (Fig. 5A), revealing a novel accumulation of MEA outside its previously reported nuclear site. In contrast, CLF and SWN were detected only in the nuclear protein enriched fraction (Fig. 5B). Further characterization of inflorescence cytoplasmic extracts by a sedimentation assay showed that both MEA and the large portion of FIE were sedimented from the soluble fraction (see Supplementary Fig. S6A), suggesting that they may take part in a large protein complex.

As FIE and MEA are known to form a high-molecular-mass complex in the nucleus (Kohler *et al.*, 2003), the presence of a similar complex in the cytoplasmic fraction was examined. In SEC analysis of cytoplasmic extract from inflorescences of gFIE-GFP transgenic plants, MEA was found to co-elute with gFIE-GFP in fractions 11–20, corresponding to complexes with an expected size of 150–400 kDa (Fig. 5C). gFIE-GFP was also detected in fractions 2–9, corresponding to large protein complexes of 600–1200 kDa (Fig. 5C), similarly to the observed complex in rosettes (Fig. 4).

As in rosettes, a parallel nuclear PRC2 complex was detected in inflorescences of gFIE-GFP plants, where FIE, gFIE-GFP and MEA were found to co-elute in fractions 1–11, which correspond to protein complexes with molecular masses of 400–1200 kDa (see Supplementary Fig. S6B).

To test whether FIE and MEA directly interact in the cytoplasm, a bimolecular fluorescence complementation assay (BiFC) (Bracha-Drori *et al.*, 2004; Walter *et al.*, 2004; Ohad *et al*., 2007) was performed in Arabidopsis, which is the endogenous environment for the examined proteins. Wild-type Arabidopsis seedlings transiently coexpressing YC-FIE and either MEA, CLF or SWN methyltransferases, fused to the YN portion, were analysed. In agreement with previous reports, FIE indeed interacted with each of the tested HMTases. FIE and MEA interaction was detected both in the nucleus and in the cytosol (Fig. 5D). On the other hand, the interaction of FIE with CLF or SWN was detected only in the nucleus (Fig. 5D), which coincides with the presence of CLF and SWN with FIE only in nuclear high molecular complexes (see Supplementary Fig. S5; Wood *et al.*, 2006). Co-immunoprecipitation experiments further demonstrated the ability of FIE and MEA to interact in the cytoplasm. Transiently expressed YN-MEA and YC-FIE proteins were precipitated together from a cytoplasmic extract (Supplementary Fig. S7A), as reported previously by Bracha-Drori *et al.* (2004). Furthermore, transgenic plants expressing MEA protein fused with 3×HA-tag (Supplementary Fig. S7B) were used in an independent co-IP assay. The MEA-3×HA protein was immunoprecipitated from inflorescence cytoplasmic extract using αHA antibody. Co-immunoprecipitation of the endogenous FIE was then detected by immunoblot analysis with the αFIE (Supplementary Fig. S7C).

## Discussion

The epigenetic role of plant Polycomb proteins in regulating gene expression has been explored intensively over the recent years using genetics, molecular biology and biochemical approaches (Butenko and Ohad, 2011; Bemer and Grossniklaus, 2012; Derkacheva and Hennig, 2014; de la Paz Sanchez *et al.*, 2015). In Arabidopsis, members of the three PRC2 complexes were shown to play a vital role in nearly every stage of the plant life cycle, as evident from the severe phenotypes of *pcg* mutants (Mozgova *et al.*, 2015). As *FIE* is a single copy gene encoding the WD-40 subunit common to all nuclear PRC2 complexes, it is expected to be indispensable for all PRC2 functions.

### FIE protein is present in the nuclei of all cells and tissues

Previous reports demonstrated that FIE has a central role in major developmental processes in Arabidopsis (Ohad *et al.*, 1996; Kiyosue *et al.*, 1999; Yadegari *et al.*, 2000; Katz *et al.*, 2004; Wood *et al.*, 2006; De Lucia *et al.*, 2008; Bouyer *et al.*, 2011; Kim *et al.*, 2012; Ikeuchi *et al.*, 2015). Accordingly, *FIE* transcripts were detected in numerous vegetative and reproductive plant tissues (Genevestigator database; Zimmermann *et al.*, 2004). In the current study, FIE protein was found in the nuclei of all examined cell types, organs, and tissues, demonstrating a wide temporal and spatial expression pattern (Fig. 1), which overlaps with the known distribution of *FIE* transcripts. Thus, this study provides additional molecular and biochemical evidence in support of the extensive role of FIE throughout the entire plant life cycle.

The functionality of the gFIE-GFP transgenic protein used in this study was demonstrated by its ability to fully complement the *fie* phenotype (Table 1 and Supplementary Fig. S2). Interestingly, the double-homozygous gFIE-GFP transgenic lines, bearing two WT endogenous and two transgenic *FIE* alleles, displayed a WT phenotype (not shown). In contrast, full or partial loss of FIE leads to a variety of developmental impairments (Ohad *et al.*, 1996; Kiyosue *et al.*, 1999; Luo *et al.*, 2000; Katz *et al.*, 2004). This is consistent with FIE playing a role as part of a multi-protein complex, where excess of an individual subunit would not interfere with the complex function, while loss of any subunit may abolish complex functionality.

### FIE localizes to the cytoplasm

The surprising outcome of this study is the discovery that FIE protein accumulates in the cytoplasm (Figs 1C and 3), distant from of its acknowledged site of action on the chromatin. The systematic examination of the subcellular localization of FIE determined that cytoplasmic accumulation of FIE is a general phenomenon in Arabidopsis, taking place in every cell where FIE is produced, and is not limited to specific tissues (Figs 1C and 3B, and Supplementary Fig. S3). Overall, cell fractionation experiments (Figs 1A and 3) together with live cell imaging (Fig. 1C and Supplementary Fig. S3) indicated that endogenous and transgenic FIE proteins localize in both the nuclear and the cytoplasmic compartments.

The presence of the typically nuclear Polycomb proteins in the cytoplasm has not been previously reported in plant cells. However, the unique cytosolic accumulation of the PRC2 components may indicate that plant PcGs have alternative non-nuclear functions. This suggestion is in agreement with accumulating evidences from studies in metazoa, demonstrating cytoplasmic localization and function of PcG proteins (Rietzler *et al.*, 1998; Ogawa *et al.*, 2003; Witte *et al.*, 2004; Su *et al.*, 2005; Bryant *et al.*, 2008; Philipp *et al.*, 2010; Follmer *et al.*, 2012; Roy *et al.*, 2012).

### FIE and MEA take part in a cytoplasmic high-molecular-mass complex

In both vegetative and floral tissues, cytoplasmic FIE was found to take part in high-molecular-mass protein complexes (Figs 4 and 5C). These findings support the suggestion that FIE has a functional role in the cytoplasm, rather than simply being detected in this compartment prior to its translocation to the nucleus. The molecular masses of these cytoplasmic FIE-containing complexes were distributed across a wide range, from 150 to 1200 kDa (Figs 4 and 5C). In both rosettes and inflorescences, cytoplasmic FIE was detected in two main peaks: one corresponding to a molecular mass of 150–500 kDa and the second corresponding to a molecular mass of ~1–1.2 MDa (Figs 4 and 5C). Therefore, it is possible that in these two plant tissues, FIE forms more than one cytoplasmic complex, each with a different protein composition. In rosette leaves, however, the majority of cytosolic FIE appeared in a monomeric form, as FIE eluted as monomers in the size-exclusion chromatography (Fig. 4), and remained in the soluble fraction during sedimentation (Fig. 3C). The significance of this phenomenon is not yet clear.

In contrast to rosettes, in inflorescences a large portion of the cytosolic FIE was found in a multiprotein complex (Supplementary Fig. S5A). This complex also included MEA, an HMTase subunit of the nuclear PRC2 complex in floral reproductive organs. The above is based on the fact that endogenous MEA protein was detected in the cytoplasmic fraction isolated from inflorescences (Fig. 5A), and co-eluted with FIE in the fractions corresponding to the 150–500 kDa complex (Fig. 5C). Moreover, direct FIE and MEA interaction in the novel cytoplasm compartment was demonstrated *in planta* via a BiFC assay in Arabidopsis leaves (Fig. 5D), and further substantiated by the ability of a tagged MEA protein to co-precipitate the endogenous FIE from cytosolic extract (see Supplementary Fig. S7C). The cytoplasmic interaction between MEA and FIE was previously observed using a BiFC assay in *N. benthamiana* leaves (Bracha-Drori *et al.*, 2004; Mosquna *et al.*, 2009), which was attributed to overexpression in a non-endogenous system. However, based on the current results using three independent approaches to demonstrate FIE and MEA endogenous localization and formation of high-molecular-mass complexes in the cytoplasm in Arabidopsis cells, it is proposed that PRC2 complexes might have cytosolic functions.

Nuclear PRC2 complexes were detected, as expected, in both rosettes and inflorescences, ranging between 150 and 1200 kDa (Supplementary Figs S5 and S6B). Nuclear FIE co-eluted with MEA in inflorescences (Supplementary Fig. S6B), and with SWN and CLF in rosettes (see Supplementary Fig. S5). In both cases, these fractions corresponded to high-molecular-mass protein complexes, including fractions larger than 600 kDa (Supplementary Fig. S5). These results are in line with previous independent reports (Kohler *et al.*, 2003; Wood *et al.*, 2006; De Lucia *et al.*, 2008). Overall, our findings indicate that nuclear and cytoplasmic PcG complexes coexist in parallel in Arabidopsis cells.

Interestingly, in the inflorescences the sizes of FIE–MEA complexes varied between the cytoplasm and the nucleus. The nuclear complex corresponded to ~500–1200 kDa (see Supplementary Fig. S6B), in contrast to the putative cytoplasmic FIE–MEA complex that ranged between 150 and 500 kDa (Fig. 5D), suggesting a qualitative and/or quantitative difference in the composition of the cytoplasmic and nuclear complexes.

### Potential role of the cytoplasmic PcG complex

The identification of plant PcG proteins in the cytoplasm raises the question of their potential non-nuclear function. Although only two PRC2 proteins were identified in the cytosol in the current study, the putative cytoplasmic PcG complex(es) may include additional core members of the nuclear PRC2, namely zinc-finger and MSI1 proteins, or any of the nearly 20 non-core PRC2 proteins identified in Arabidopsis (Pikaard and Mittelsten Scheid, 2014). This suggestion is based on several independent studies in metazoa that demonstrated the presence of multiple PRC2 components in the cytoplasm of different cell types. In *Drosophila*, nine PcG proteins, including the core PRC2 subunits E(z), Su(12)z, and PSC, were identified in the cytoplasm of interphase-stage S2 embryonic cells (Follmer *et al.*, 2012). Moreover, the PRC2 subunits EZH1 and EZH2 (HMTases), Su(z)12 (zinc finger) and EED (FIE homolog) were detected in the cytoplasm of various mammalian cells (Rietzler *et al.*, 1998; Ogawa *et al.*, 2003; Su *et al.*, 2005; Bryant *et al.*, 2008). It would thus be interesting to determine the composition of the plant cytoplasmic FIE-containing complexes in vegetative and reproductive tissues, and compare them with nuclear PRC2 complexes.

In contrast to the wide accumulation pattern of FIE in Arabidopsis, MEA protein was so far detected only in reproductive tissues (Kohler *et al.*, 2003; Jullien *et al.*, 2006). The putative cytoplasmic FIE–MEA-containing PRC2 complex is therefore expected to be limited to specific cells in developing ovules and seeds. It was reasonable to assume that analogous cytoplasmic complexes in vegetative tissues would contain either of the two remaining HMTases, namely CLF or SWN, as the catalytic subunit. However, in contrast to MEA, localization of CLF and SWN appeared to be strictly nuclear (Fig. 5A, B). It is thus unclear whether the cytosolic FIE-containing complexes detected in rosette leaves are genuine PRC2 complexes that contain a methyltransferase subunit. Alternatively, in the vegetative organs, cytoplasmic FIE may interact with non-PcG proteins to form a completely new type of complex.

Protein function can be elucidated from the observation of mutant phenotype; however, no specific cytosolic function is known to be associated with *fie* and *mea* KO mutant phenotypes. Given the functional conservation of the nuclear PcG function between plant and animal kingdoms, it is possible that such conservation exists for the cytoplasmic functions as well. In metazoan cells, various PRC2 subunits were shown to function in the cytosol downstream of activated growth-factor receptors, to promote proliferation, differentiation and dynamic cytoskeletal responses (Ogawa *et al.*, 2003; Su *et al.*, 2005; Bryant *et al.*, 2008; Philipp *et al.*, 2010; Roy *et al.*, 2012). Mammalian EED and EZH2 proteins actively translocate from the nucleus to cytoplasm in response to stimulation from plasma membrane tumor necrosis factor (EED; Philipp *et al.*, 2010), integrin (EED; Witte *et al.*, 2004) and Notch receptors (EZH2; Roy *et al.*, 2012). Notably, a catalytically active Polycomb complex containing the nuclear PRC2 subunits EZH2, EED and Su(z)12 was isolated from a cytoplasmic fraction of mammalian T cells and fibroblasts. The lysine-methylating activity of this complex was shown to be crucial for receptor-induced actin reorganization and proliferation (Su *et al.*, 2005). Most of the direct interactors of mammalian cytosolic PRC2 that take part in regulating actin organization (Ogawa *et al.*, 2003; Su *et al.*, 2005; Roy *et al.*, 2012), such as the GTP/GDP exchange factor Vav1, do not have orthologs in Arabidopsis. However the mammalian receptor for activated C kinase 1 (RACK1), which was shown to interact with EED in the cytoplasm (Philipp *et al.*, 2010), has homologs in Arabidopsis, which act in a signal transduction pathway similar to their mammalian counterparts (Islas-Flores *et al.*, 2015). Hence, it would be interesting to test whether the Arabidopsis cytoplasmic FIE or FIE–MEA-containing complexes can interact with cytoplasmic components, such as the actin-regulating cellular machinery, or play roles in signal transduction pathways in a similar manner to that reported in mammalian cells, though not necessarily through the conserved homologous components.

## Supplementary data

Supplementary data are available at *JXB* online.

Figure S1. Characterization of PRC2 specific antibodies.

Figure S2. Morphology of gFIE-GFP complemented *fie-1*^*–/–*^ plants is indistinguishable from WT plants.

Figure S3. gFIE-GFP protein accumulates in all Arabidopsis tissues and organs.

Figure S4. Endogenous FIE and gFIE-GFP proteins are present in the cytoplasm.

Figure S5. Nuclear FIE complexes in vegetative tissues of Arabidopsis plant.

Figure S6. FIE and MEA in reproductive tissues of Arabidopsis plant.

Figure S7. FIE and MEA proteins co-immunoprecipitation from cytoplasmic fraction.

Supplementary Data
