# Supplementary material for: FIE, a nuclear PRC2 protein, forms cytoplasmic complexes in Arabidopsis thaliana
Source: J Exp Bot. 2016 Oct 17;67(21):6111–23. doi: 10.1093/jxb/erw373 (PMC5100023; doi:10.1093/jxb/erw373)
Supplement: Supplementary Data [file supp_67_21_6111__index.html]

FIE, a nuclear PRC2 protein, forms cytoplasmic complexes in Arabidopsis thaliana — FIE, a nuclear PRC2 protein, forms cytoplasmic complexes in Arabidopsis thaliana — Supplementary Data 

# FIE, a nuclear PRC2 protein, forms cytoplasmic complexes in *Arabidopsis thaliana*

## Supplementary Data

Data files

- supplementary\_figures\_S1\_S7.pdf - Supplementary Data
