# Supplementary material for: FIE, a nuclear PRC2 protein, forms cytoplasmic complexes in Arabidopsis thaliana
Source: J Exp Bot. 2016 Oct 17;67(21):6111–23. doi: 10.1093/jxb/erw373 (PMC5100023; doi:10.1093/jxb/erw373)
Supplement: Supplementary Data [file supp_erw373_supplementary_figures_S1_S7.pdf]

FIE, a nuclear PRC2 protein, forms cytoplasmic complexes in *Arabidopsis thaliana*

Moran Oliva [1,2,6,*]., Yana Butenko [1,3,*]., Tzung-Fu Hsieh [4]., Ofir Hakim [1,5]., Aviva Katz [1]., Nechama I. Smorodinsky [7]., Daphna Michaeli [1]., Robert L. Fischer [8]., and Nir Ohad [1,9,†].

Supplemental Figures

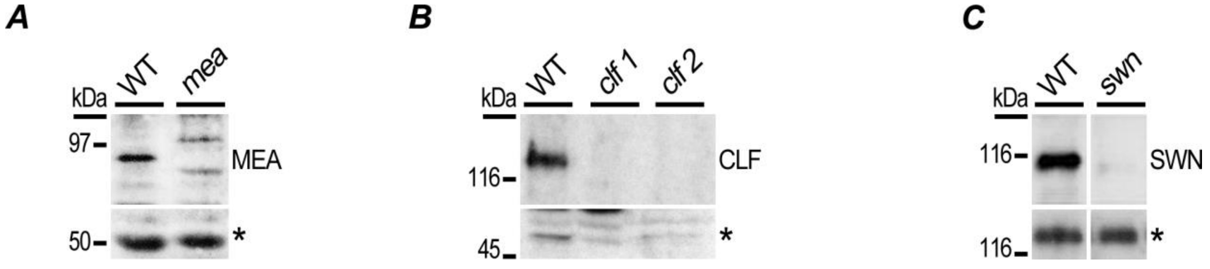

Supplementary Figure S1. Characterization of anti-PRC2 antibodies specificity. (**A**) αMEA antibody identifies a 97 kDa polypeptide in inflorescence cytoplasmic extract from WT plant, but not in *mea* homozygous mutant. 100 µg total protein loaded in each lane. Equal loading was based on the presence of non-specific low-molecular-weight proteins in WT and mutant (marked with asterisk). (**B**) αCLF antibody identifies a ~130 kDa polypeptide in extracts of nuclear proteins from rosette leaves of WT *Arabidopsis,* but not in two *clf* homozygous mutant lines. *clf 1* mutant line: CS8853 line (*clf-2* allele (Goodrich et al., 1997)); *clf 2* mutant line: Salk_006658 line. Equal loading was based on the presence of non-specific low-molecular-weight proteins detected in WT and mutant (marked with asterisk). (**C**) αSWN antibody identifies a ~110 kDa polypeptide in extracts of nuclear proteins from rosette leaves of WT *Arabidopsis,* but not in *swn* homozygous mutant. Equal loading was based on the presence of CLF protein in WT and mutant, detected with αCLF (marked with asterisk).

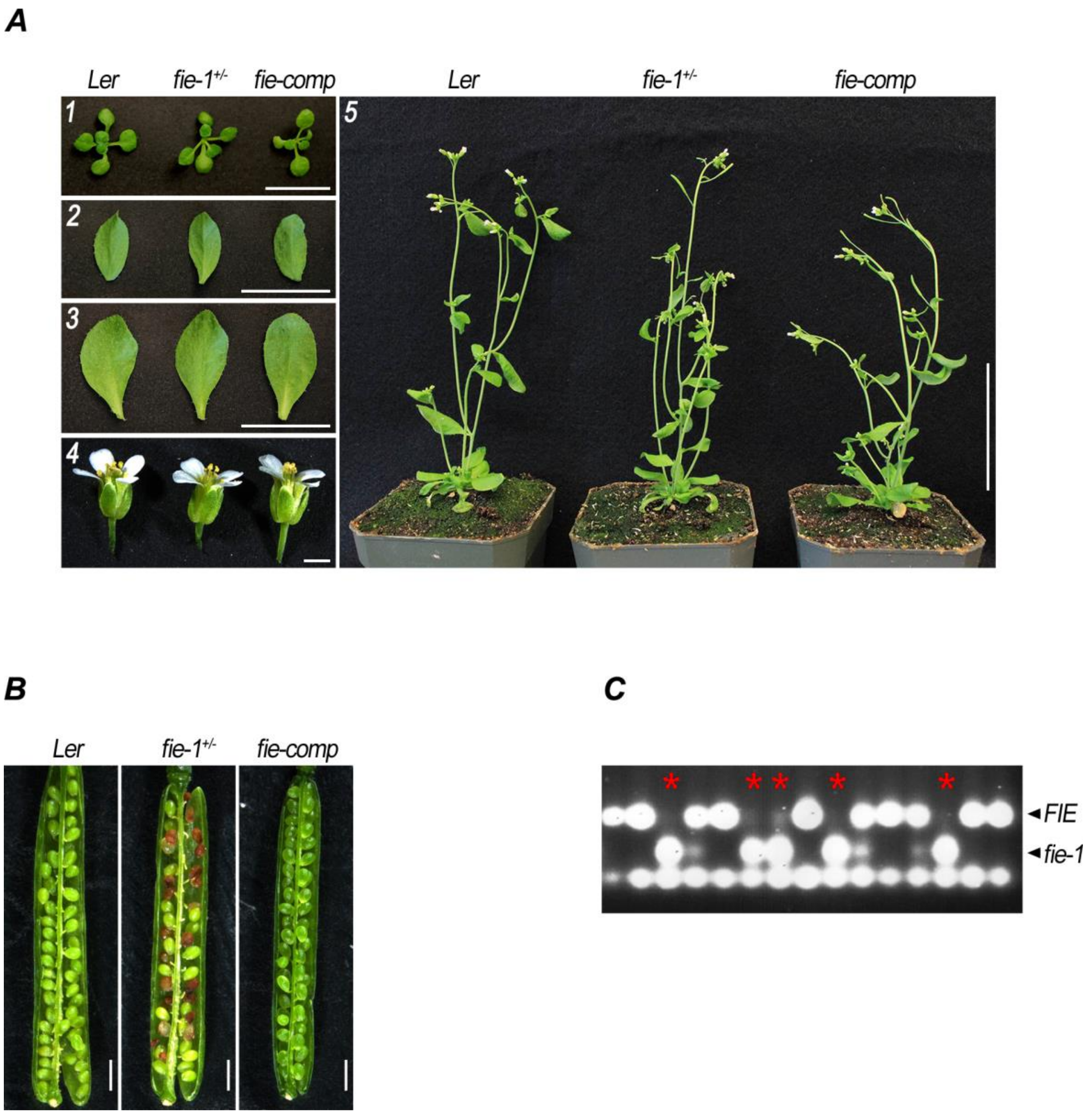

Supplementary Figure S2. Morphology of gFIE-GFP complemented *fie-1*$^{-/-}$ plants is indistinguishable from WT plants. (**A-B**) Wild-type Landsberg *erecta* (*Ler*) plants, *fie-1* heterozygotes and progenies of the line #142 that are homozygous *fie-1*$^{-/-}$ *gFIE-GFP*$^{+/+}$ (*fie-comp*) were documented along the plant life. (**A**) Seedling to mature plants: (*1*) Seedlings at four true leaves stage, S.b: 1 cm; (*2*) Cauline leaves of adult plants, S.b: 1 cm; (*3*) Rosette leaves of adult plants, S.b: 1 cm; (*4*) Flowers, S.b: 1 mm; (*5*) One month old plants. S.b: 5 cm. (**B**) Representing siliques of *Ler*, *fie-1* heterozygotes and *fie-comp* plants, S.b: 1 mm. Siliques were examined 40 days after germination, 8-10 siliques were examined from three independent plants of each genotype. *fie-1*$^{+/-}$ siliques demonstrated a typical ~50% seed abortion (normal 482, aborted 437; distributing - normal: 126, 190, 166; aborted 125, 157, 155), as reported by (Ohad *et al.*, 1996). No seed abortions were observed in *fie-comp* plants (normal 1140, aborted 1; distributing - normal 390, 352, 398; aborted 0, 0, 1) or in *Ler* plants (normal 1147, aborted 0; distributing - normal 401, 339, 407; aborted 0, 0, 0) indicating that gFIE-GFP can fully complement *fie-1*mutation.; (**C**) Genotyping via PCR showing progenies of line #142 homozygous for *fie-1*.

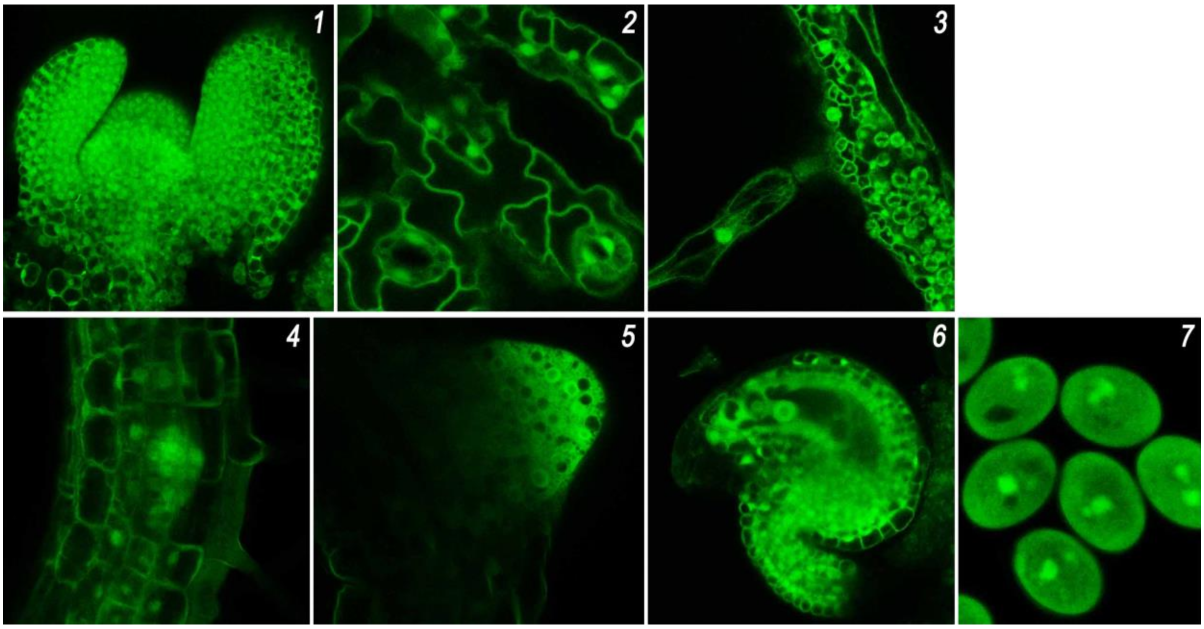

Supplementary Figure S3. gFIE-GFP protein accumulates in all *Arabidopsis* tissues and organs. *gFIE-GFP* epifluorescence was examined in reproductive and vegetative tissues and organs in an independent *ProFIE:FIE*$_{gDNA}$*-GFP* line designated DM_633. Each image was taken from a single focal plane. (*1*) SAM of two weeks old seedling with the adjacent newly formed leaves; (*2*) Rosette leaf, abaxial epidermis; (*3*) Trichome on the surface of a rosette leaf; (*4*) Initial of branching root; (*5*) Budding lateral root; (*6*) Female gametophyte; (*7*) Pollen.

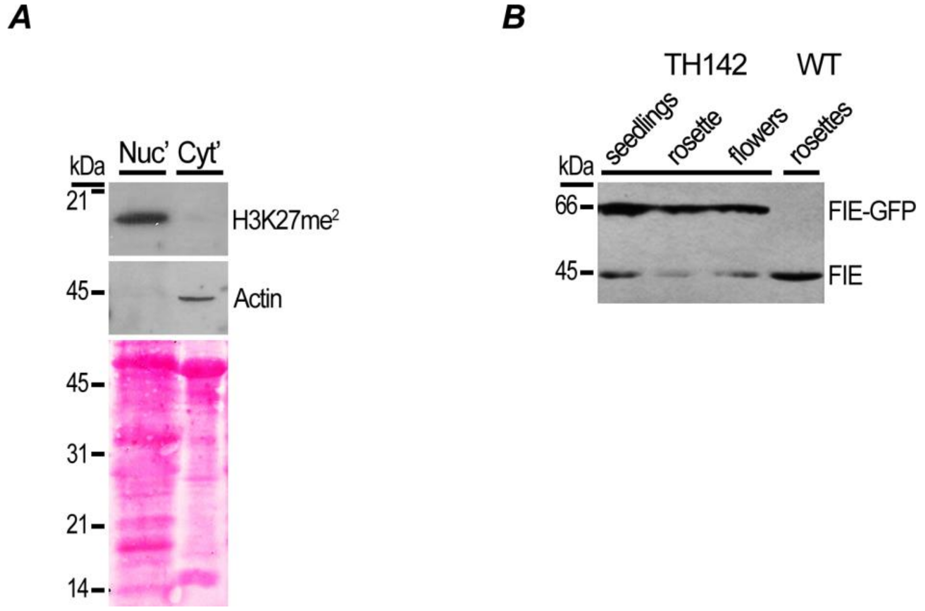

Supplementary Figure S4. Endogenous FIE and gFIE-GFP proteins are present in the cytoplasm. (**A**) The purity of the nuclei-enriched (Nuc') and cytoplasmic (Cyt') fractions obtained by differential extraction methods was assessed by immunoblotting with αH3K27me^2 (Upstate, 07452) and α-actin (1:5000, MP Biomedicals). Ponceau staining was used to assess equal loading of samples. (**B**) Detection of native FIE and transgenic gFIE-GFP protein in *Arabidopsis* tissues. Similar volumes of cytosolic protein extracts from different tissues of TH142 (*ProFIE:FIE*$_{gDNA}$*-GFP*) and WT plants were separated by SDS-PAGE and immune detected using αFIE antibodies.

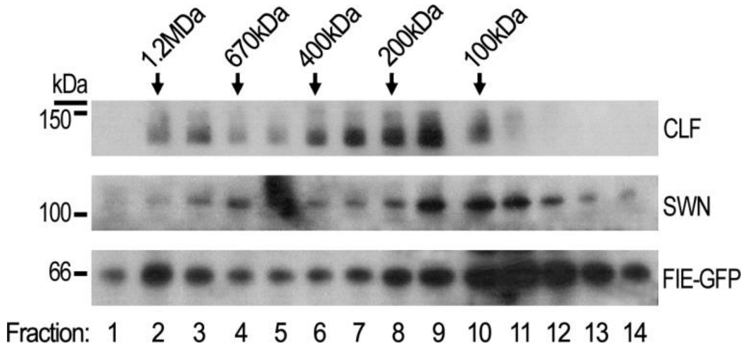

Supplementary Figure S5. Nuclear FIE complexes in vegetative tissues of *Arabidopsis* plant. Nuclei-enriched fraction was isolated from rosette leaf tissues of pre-bolted *ProFIE:FIE*$_{gDNA}$*-GFP* plants. The nuclei were lysed in native conditions and the extracted proteins were separated on a gel-filtration Superose 6 column. The obtained fractions (1 ml) were probed using αCLF, αSWN and αGFP antibodies (top to bottom panel). Size markers (kDa) are indicated above relevant fractions.

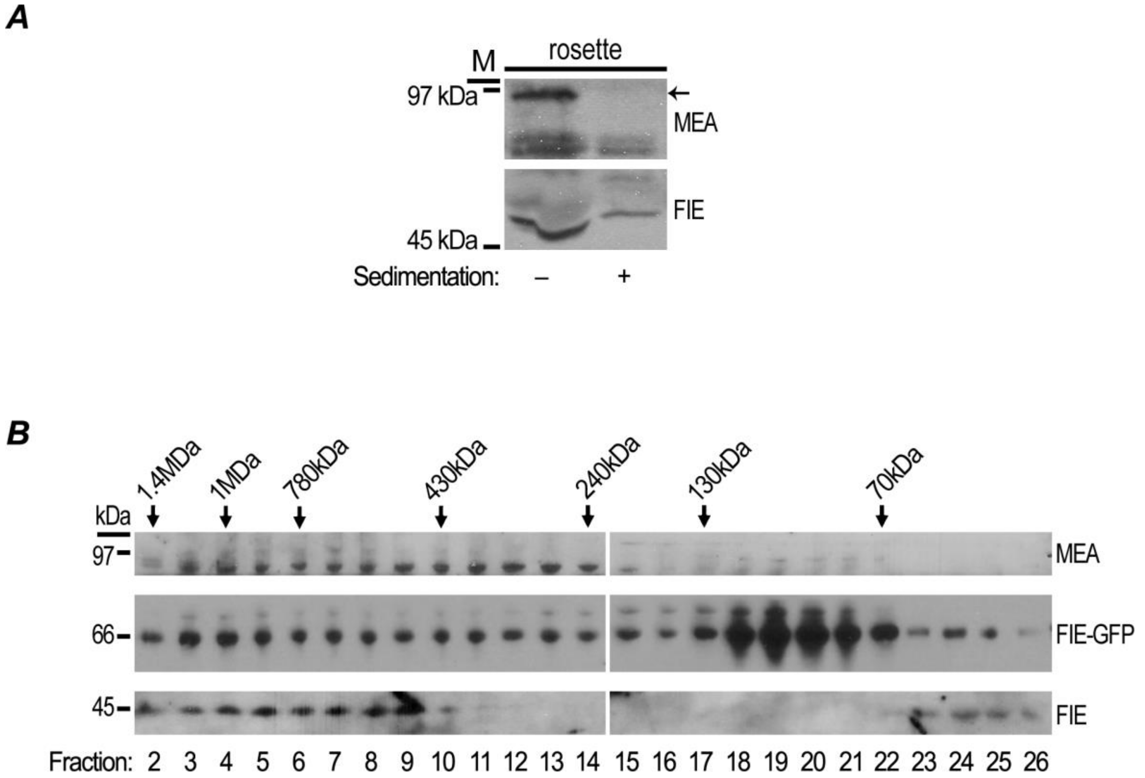

Supplementary Figure S6. FIE and MEA are present in complexes in reproductive tissues of *Arabidopsis* plant. (**A**) Sedimentation assay of native cytosolic protein extracts from inflorescence tissues of WT plants. The supernatant soluble fraction was analyzed by SDS-PAGE and probed using αFIE and αMEA antibodies. Migration of FIE protein in the non-sedimented fraction, to an apparent lower molecular size band, may result from the presence of large amount of RuBisCO large chain (~53 kDa). (**B**) Nuclei-enriched fraction was isolated from inflorescences of *ProFIE:FIE*$_{gDNA}$*-GFP* plants. The nuclei were lysed in non-denaturative conditions and the extracted proteins were separated on a size-exclusion chromatography Superose 6 column (GE Healthcare Life Sciences). The obtained fractions (0.5 ml) were probed using αMEA, αFIE and αGFP antibodies (top to bottom panel). Size markers (kDa) are indicated above relevant fractions.

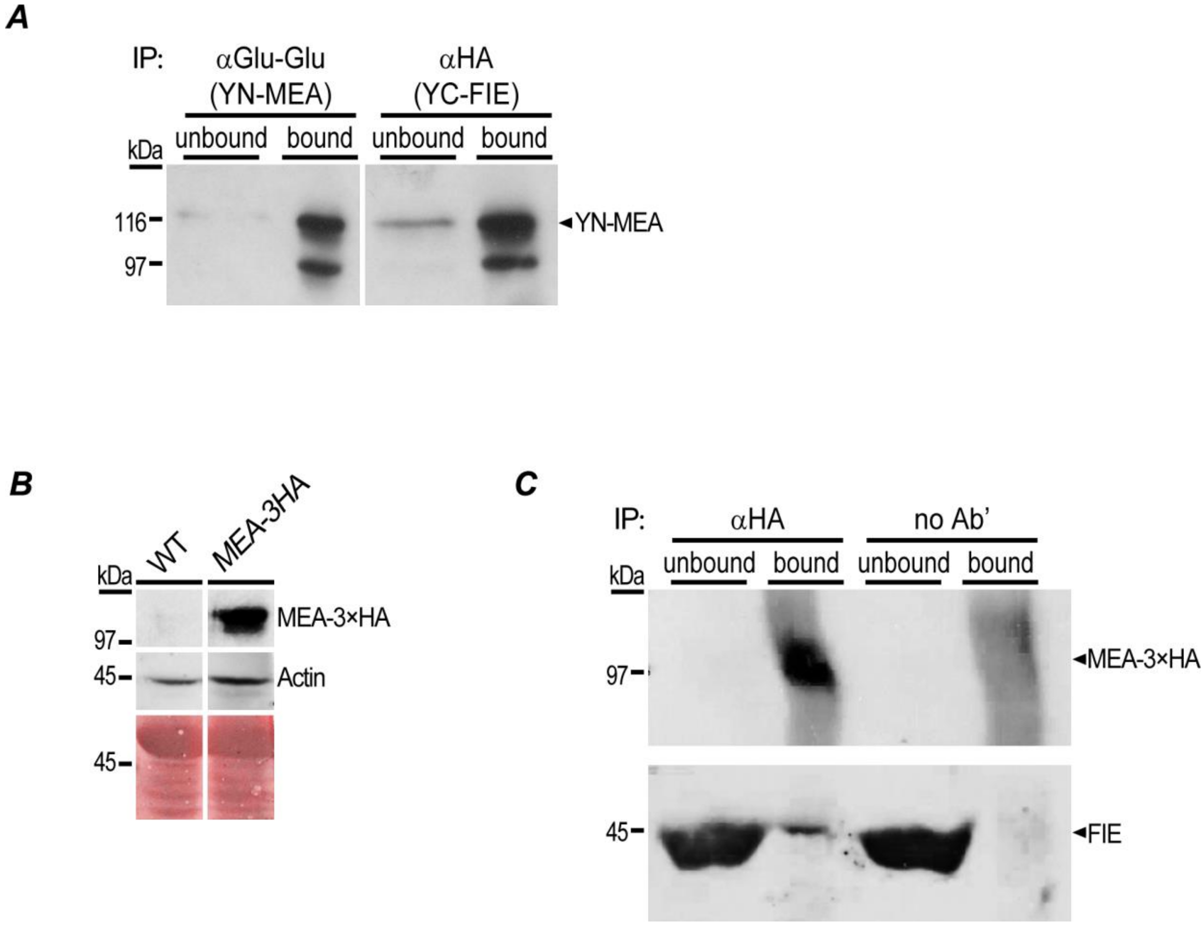

Supplementary Fig S7. FIE and MEA proteins co-immunoprecipitate in cytoplasmic fraction. (**A**) Cytoplasmic fraction was isolated from *N. benthamiana* leaves transiently expressing YC-HA-FIE and YN-GG-MEA fusion proteins. Immunocomplexes were precipitated with αHA or αGlu-Glu antibodies. The blots were probed with αGFP antibody that detects the YN-tag. Bound – precipitated immunocomplexes, unbound – nonprecipitated proteins. Input was loaded with 1/30 of the original native extract volume used for immunocomplex precipitation. (**B**-**C**) Co-immunoprecipitation of endogenous FIE with MEA-3×HA. (**B**) MEA-3×HA transgenic protein is recognized at its expected size by αHA antibody in cytoplasmic extracts from inflorescences of MEA-3HA plants. Extraction of cytoplasmic proteins was validated by immunoblotting with α-actin (1:1000, MP Biomedicals). CLF protein, which we and others have shown to localize to the nucleus (See Fig. 5B and D), was not detected by αCLF antibody in this fraction (not shown). Ponceau staining was used to assess equal loading of samples. (**C**) Cytoplasmic fraction was isolated from inflorescences of MEA-3HA plants and immunocomplexes were precipitated with αHA antibody. The blots were probed with αHA (upper panel) and αFIE (lower panel). Bound – precipitated immunocomplexes, unbound – nonprecipitated proteins. Input was loaded with 1/30 of the original native extract volume used for immunocomplex precipitation. Arrowheads in A and C point to MEA and FIE proteins.
